# Novel Anticancer Strategies II

**DOI:** 10.3390/pharmaceutics15020605

**Published:** 2023-02-10

**Authors:** Hassan Bousbaa

**Affiliations:** 1 UNIPRO-Oral Pathology and Rehabilitation Research Unit, Institute of Health Sciences (IUCS), Cooperativa de Ensino Superior Politécnico e Universitário (CESPU), Rua Central de Gandra 1317, 4585-116 Gandra, Portugal; hassan.bousbaa@iucs.cespu.pt; 2 Centro Interdisciplinar de Investigação Marinha e Ambiental (CIIMAR), Universidade do Porto, Terminal de Cruzeiros do Porto de Leixões, Av. General Norton de Matos s/n, 4450-208 Matosinhos, Portugal

Owing to the exceptional complexity of the development and progression of cancer, diverse cancer types are alarmingly increasing worldwide. Researchers and clinicians are facing a unique challenge. Radiotherapy and chemotherapy continue to be the main therapeutic options. However, these conventional therapies are associated with undesirable toxicity and resistance, and, as such, have proved unsuccessful in eradicating tumors completely. Consequently, the search for new anticancer drugs and novel drug delivery strategies are urgently needed to overcome conventional therapeutics’ drawbacks and, hopefully, to offer more effective therapeutic options. This Special Issue (https://www.mdpi.com/journal/pharmaceutics/special_issues/novel_anticancer_volume_II, (accessed on 31 January 2023)) was created after the successful first volume (https://www.mdpi.com/journal/pharmaceutics/special_issues/novel_anticancer, (accessed on 28 January 2023)), and is dedicated to innovative research on the development and validation of novel anticancer approaches, hopefully with relevant clinical value [1].

Sixteen original articles and fifteen reviews were published that provide the state-of-the-art of novel anticancer approaches. This editorial briefly summarizes the findings and highlights derived from the published articles.

Cancer immunotherapy deploys the immune system as a tool to treat neoplastic disease, and it is now firmly established as a novel pillar of cancer care. In this regard, Cao et al. nicely reviewed immunotherapy for triple-negative breast cancer [2]. The paper by Zha and colleagues used engineered cell membrane-derived programmed death-ligand 1(PD-1) nanovesicles to encapsulate low-dose gemcitabine (PD-1&GEM NVs) to show that PD-1&GEM NVs could synergistically inhibit the proliferation of triple-negative breast cancer. The study highlighted the potential of the tested combination in the nanovesicles for triple-negative breast cancer therapy [3]. In their review paper, Pao et al. discussed recent progress in neoantigen identification and applications for cancer vaccines and summarized the results of ongoing trials [4].

Molecular-targeted therapies interfere with specific molecules to block cancer growth and progression, with the advantages of high efficiency, few side effects, and low drug resistance for patients. Cell signaling and cell cycle components are involved in many aspects of cancer cell proliferation and survival and, thus, are primary targets for cancer therapy [5]. As such, several inhibitors against mitotic regulation and surveillance components have been developed, with promising outcomes in preclinical assays. Unfortunately, these new antimitotics exhibited limited efficacy as monotherapy in clinical trials, as nicely reviewed in [6]. In a goal to give a second chance to these antimitotics, Pinto et al. combined the antimitotic BI2536, a potent inhibitor of Polo-like kinase 1 (PLK1), with Navitoclax, a BH3-mimetic and apoptosis inducer. The combination showed synergy in lung cancer cell-killing activity in 2D and 3D cell culture systems [7]. As to mitotic surveillance components, Silva and Bousbaa reviewed the structure and function of the spindle assembly checkpoint gene BUB3, its expression in cancer, its association with survival prognoses, and its potential as an anticancer target [8]. Other molecular targets are multifunctional enzymes, which have been proposed as promising drug targets for cancer therapy. Teixeira and Sousa reviewed the structure and functions of four multifunctional enzymes, the inhibition of which has already demonstrated promising anti-cancer effects [9]. As a solution to undruggable targets, the review by Bartolucci et al. described the oligonucleotide therapeutics targeting RNA or DNA sequences as an emerging class of precision anticancer biotherapeutics [10]. Other papers reported molecular-targeted therapy strategies to block cancer growth and progression [11,12].

Cancer eradication is often compromised by a small population of Cancer Stem Cells (CSCs) within tumors, with capabilities of self-renewal, differentiation, and tumorigenicity, thereby causing tumor relapses. CSCs are also known for their therapy resistance. For instance, as reviewed by Quiroz-Reyes et al., CSCs can quickly develop adaptative evasion mechanisms for Tumor necrosis factor (TNF)-related apoptosis-inducing ligand (TRAIL) apoptosis [13]. Several CSC biomarkers have been identified with useful applications to diagnosis, therapy, and prognosis. The paper by Morel et al. described the isolation of a subpopulation of the adrenocortical carcinoma (ACC) cell line H295R overexpressing the cell surface Hedgehog receptor Patched (Ptch1) [14]. Ptch1 is overexpressed in many cancer types and was shown to contribute to the resistance to chemotherapy in ACC. The authors showed that this cell subpopulation is more tumorigenic than the parental cells, suggesting a cancer stem cell-like phenotype, which could be responsible for the therapy resistance, relapse, and metastases in ACC patients. In another paper by Fu et al., the α5-nicotinic acetylcholine receptor (CHRNA5), previously implicated in tumor progression, was reported to contribute to hepatocellular carcinoma (HCC) progression by regulating Yes-associated protein (YAP), the key transcription factor of the Hippo pathway [15]. Interestingly, CHRNA5 promoted the stemness of HCC by regulating stemness-associated genes, such as Nanog, Sox2, and OCT, suggesting a pivotal role in the progression and drug resistance of HCC.

Several drug-delivery systems have been successfully applied in cancer therapy. The rationale behind nanocarrier systems is to deliver cytotoxic drugs to the target cells in order to reduce their overall toxicity and increase their effectiveness and selectivity. Bukhari et al. provided a comprehensive and updated review of recent research in the field of lipidic nanocarriers loaded with theranostics (therapeutic and diagnostic agents), highlighting the main strengths and potential limitations of pretargeting theranostics. Simultaneous delivery of imaging (with contrasting agents), targeting (with biomarkers), and anticancer agents by one lipidic nanocarrier system (as cancer theranostics) are becoming popular, but significant hurdles in their clinical translation remain [16]. In another paper, Javet et al. reviewed the most recent advancements in the field of nanoerythrosomes [17]. Nanoerythrosomes are red blood cell-based nanocarrier systems and are viewed as excellent and biocompatible nanoplatforms for drug delivery of various drugs, particularly antineoplastic drugs. Montaseri et al. reviewed the application of inorganic nanoparticles in photodynamic therapy (PDT) [18]. PDT involves light-sensitive medicine and a light source to destroy a targeted tumor, with either a photosensitizer or photochemotherapeutic agent localized within it. Cell-penetrating peptides (CPPs) are short peptides with intrinsic properties to deliver therapeutic molecules to cells and tissues in a nontoxic manner. In their paper, Stiltner et al. provided a comprehensive state-of-the-art review of the role of these promising peptides in cancer diagnostics and therapeutics [19]. MicroRNA (miRNA) has been identified as a good target for cancer treatment. Shadab Md and colleagues discussed the potential use of miRNA in cancer therapy and provided a detailed description of nanocarrier-based drug delivery systems to deliver miRNAs [20]. Gabriele et al. showed that the coating of melanin nanoparticles, otherwise known to be biologically benign to human cells with glucose enhanced their uptake with cancer cells cultured in low glucose concentrations, making them more susceptible to killing by laser illumination [21]. Other original papers published in this Special Issue reported drug delivery systems that have been improved to potentially enhance the effectiveness of complex cancer treatments [22,23,24,25,26].

Improving the chemical and physical properties of anticancer drugs is key to improving treatment efficacy. In their study, Kondo et al. synthesized 3-borono-l-phenylalanine (3-BPA), a positional isomer of 4-BPA, with improved water solubility, making it a possible 4-BPA substitute in future boron neutron capture therapy (BNCT) [27]. BCCT is based on the nuclear reaction that results from boron-10 irradiation with neutrons of the appropriate energy to produce high-energy alpha particles and recoiling lithium-7 nuclei. Domínguez-Jurado et al. reported the synthesis of a ruthenium compound, namely *Ru3*, that showed cytotoxic activity against breast cancer cells, which might serve as the basis for the design of more active and less toxic antitumoral compounds [28]. Drug repurposing is an efficient and economical approach to identifying novel therapeutic agents from the existing FDA-approved clinically used drug molecules. Due to the similarities between cancer immune response and the coronavirus disease 2019 (COVID-19), a list of drugs that have been approved for cancer indication by the US FDA has entered clinical trials for COVID-19 treatments [29]. In the same line of thought, ester derivatives of menahydroquinone-4, used for osteoporosis treatment, were shown to exert strong growth-inhibitory effects on all-trans retinoic acid (ATRA)-resistant acute promyelocytic leukemia cells [30].

Diagnostic findings are critical for clinical decision-making in health care. In this regard, Kejík et al. discussed how circulating tumor cell count and targeting can influence the chemotherapeutic efficacy in non-small-cell lung carcinoma (NSCLC), providing a tool for prognosis and therapy design in NSCLC [31].

Three-dimensional (3D) cell culture systems (spheroids) have gained broader use in preclinical cancer research due to their ability to mimic the structural complexity of the tissue microenvironment of the real tumor. Quarta et al. presented a reliable 3D culture system based on collagen I-blended agarose hydrogel [32]. They showed that variation in the agarose percentage affects the physical and mechanical properties of the resulting hydrogel, making it a reliable biomimetic matrix for the growth of 3D cell structures.

Today, clinicians have a wide variety of effective cancer treatments at their disposal, which has translated into a steady increase in the survival of cancer patients. Yet, cancer incidence continues to rise, posing a continuous challenge to discovering and developing better anticancer treatments, stressing the need for more (focused) cancer research. Discovering new anticancer agents, refining our understanding of the old ones, and the main ways to target them should be thoroughly explored. The works published in this Special Issue have contributed to these goals by providing insights into some promising drugs, drug targets, drug combinations, and drug delivery.

## References

[1] Bousbaa H. (2021). Novel Anticancer Strategies. Pharmaceutics.

[2] Cao Y., Chen C., Tao Y., Lin W., Wang P. (2021). Immunotherapy for Triple-Negative Breast Cancer. Pharmaceutics.

[3] Zha H., Xu Z., Xu X., Lu X., Shi P., Xiao Y., Tsai H.-I., Su D., Cheng F., Cheng X. (2022). PD-1 Cellular Nanovesicles Carrying Gemcitabine to Inhibit the Proliferation of Triple Negative Breast Cancer Cell. Pharmaceutics.

[4] Pao S.-C., Chu M.-T., Hung S.-I. (2022). Therapeutic Vaccines Targeting Neoantigens to Induce T-Cell Immunity against Cancers. Pharmaceutics.

[5] Kashyap D., Garg V.K., Sandberg E.N., Goel N., Bishayee A. (2021). Oncogenic and Tumor Suppressive Components of the Cell Cycle in Breast Cancer Progression and Prognosis. Pharmaceutics.

[6] Novais P., Silva P.M.A., Amorim I., Bousbaa H. (2021). Second-Generation Antimitotics in Cancer Clinical Trials. Pharmaceutics.

[7] Pinto B., Novais P., Henriques A.C., Carvalho-Tavares J., Silva P.M.A., Bousbaa H. (2022). Navitoclax Enhances the Therapeutic Effects of PLK1 Targeting on Lung Cancer Cells in 2D and 3D Culture Systems. Pharmaceutics.

[8] Silva P.M.A., Bousbaa H. (2022). BUB3, beyond the Simple Role of Partner. Pharmaceutics.

[9] Teixeira C.S.S., Sousa S.F. (2021). Current Status of the Use of Multifunctional Enzymes as Anti-Cancer Drug Targets. Pharmaceutics.

[10] Bartolucci D., Pession A., Hrelia P., Tonelli R. (2022). Precision Anti-Cancer Medicines by Oligonucleotide Therapeutics in Clinical Research Targeting Undruggable Proteins and Non-Coding RNAs. Pharmaceutics.

[11] Soond S.M., Savvateeva L.V., Makarov V.A., Gorokhovets N.V., Townsend P.A., Zamyatnin A.A. (2021). Cathepsin S Cleaves BAX as a Novel and Therapeutically Important Regulatory Mechanism for Apoptosis. Pharmaceutics.

[12] Leis-Filho A.F., de Faria Lainetti P., Emiko Kobayashi P., Fonseca-Alves C.E., Laufer-Amorim R. (2021). Effects of Lapatinib on HER2-Positive and HER2-Negative Canine Mammary Carcinoma Cells Cultured In Vitro. Pharmaceutics.

[13] Quiroz-Reyes A.G., Delgado-Gonzalez P., Islas J.F., Gallegos J.L.D., Martínez Garza J.H., Garza-Treviño E.N. (2021). Behind the Adaptive and Resistance Mechanisms of Cancer Stem Cells to TRAIL. Pharmaceutics.

[14] Feliz Morel Á.J., Hasanovic A., Morin A., Prunier C., Magnone V., Lebrigand K., Aouad A., Cogoluegnes S., Favier J., Pasquier C. (2022). Persistent Properties of a Subpopulation of Cancer Cells Overexpressing the Hedgehog Receptor Patched. Pharmaceutics.

[15] Fu Y., Ci H., Du W., Dong Q., Jia H. (2022). CHRNA5 Contributes to Hepatocellular Carcinoma Progression by Regulating YAP Activity. Pharmaceutics.

[16] Bukhari S.I., Imam S.S., Ahmad M.Z., Vuddanda P.R., Alshehri S., Mahdi W.A., Ahmad J. (2021). Recent Progress in Lipid Nanoparticles for Cancer Theranostics: Opportunity and Challenges. Pharmaceutics.

[17] Javed S., Alshehri S., Shoaib A., Ahsan W., Sultan M.H., Alqahtani S.S., Kazi M., Shakeel F. (2021). Chronicles of Nanoerythrosomes: An Erythrocyte-Based Biomimetic Smart Drug Delivery System as a Therapeutic and Diagnostic Tool in Cancer Therapy. Pharmaceutics.

[18] Montaseri H., Kruger C.A., Abrahamse H. (2021). Inorganic Nanoparticles Applied for Active Targeted Photodynamic Therapy of Breast Cancer. Pharmaceutics.

[19] Stiltner J., McCandless K., Zahid M. (2021). Cell-Penetrating Peptides: Applications in Tumor Diagnosis and Therapeutics. Pharmaceutics.

[20] Md S., Alhakamy N.A., Karim S., Gabr G.A., Iqubal M.K., Murshid S.S.A. (2021). Signaling Pathway Inhibitors, MiRNA, and Nanocarrier-Based Pharmacotherapeutics for the Treatment of Lung Cancer: A Review. Pharmaceutics.

[21] Gabriele V.R., Mazhabi R.M., Alexander N., Mukherjee P., Seyfried T.N., Nwaji N., Akinoglu E.M., Mackiewicz A., Zhou G., Giersig M. (2021). Light- and Melanin Nanoparticle-Induced Cytotoxicity in Metastatic Cancer Cells. Pharmaceutics.

[22] Singh B., Shukla N., Kim J., Kim K., Park M.-H. (2021). Stimuli-Responsive Nanofibers Containing Gold Nanorods for On-Demand Drug Delivery Platforms. Pharmaceutics.

[23] Puiu R.A., Balaure P.C., Constantinescu E., Grumezescu A.M., Andronescu E., Oprea O.-C., Vasile B.S., Grumezescu V., Negut I., Nica I.C. (2021). Anti-Cancer Nanopowders and MAPLE-Fabricated Thin Films Based on SPIONs Surface Modified with Paclitaxel Loaded β-Cyclodextrin. Pharmaceutics.

[24] Nistorescu S., Udrea A.-M., Badea M.A., Lungu I., Boni M., Tozar T., Dumitrache F., Maraloiu V.-A., Popescu R.G., Fleaca C. (2021). Low Blue Dose Photodynamic Therapy with Porphyrin-Iron Oxide Nanoparticles Complexes: In Vitro Study on Human Melanoma Cells. Pharmaceutics.

[25] van Zundert I., Bravo M., Deschaume O., Cybulski P., Bartic C., Hofkens J., Uji-i H., Fortuni B., Rocha S. (2021). Versatile and Robust Method for Antibody Conjugation to Nanoparticles with High Targeting Efficiency. Pharmaceutics.

[26] Studenovský M., Rumlerová A., Kovářová J., Dvořáková B., Sivák L., Kostka L., Berdár D., Etrych T., Kovář M. (2022). HPMA Copolymer Mebendazole Conjugate Allows Systemic Administration and Possesses Antitumour Activity In Vivo. Pharmaceutics.

[27] Kondo N., Hirano F., Temma T. (2022). Evaluation of 3-Borono-l-Phenylalanine as a Water-Soluble Boron Neutron Capture Therapy Agent. Pharmaceutics.

[28] Domínguez-Jurado E., Cimas F.J., Castro-Osma J.A., Juan A., Lara-Sánchez A., Rodríguez-Diéguez A., Shafir A., Ocaña A., Alonso-Moreno C. (2021). Tuning the Cytotoxicity of Bis-Phosphino-Amines Ruthenium(II) Para-Cymene Complexes for Clinical Development in Breast Cancer. Pharmaceutics.

[29] Costa B., Vale N. (2021). A Review of Repurposed Cancer Drugs in Clinical Trials for Potential Treatment of COVID-19. Pharmaceutics.

[30] Yamakawa H., Setoguchi S., Goto S., Watase D., Terada K., Nagata-Akaho N., Toki E., Koga M., Matsunaga K., Karube Y. (2021). Growth Inhibitory Effects of Ester Derivatives of Menahydroquinone-4, the Reduced Form of Vitamin K2(20), on All-Trans Retinoic Acid-Resistant HL60 Cell Line. Pharmaceutics.

[31] Kejík Z., Kaplánek R., Dytrych P., Masařík M., Veselá K., Abramenko N., Hoskovec D., Vašáková M., Králová J., Martásek P. (2021). Circulating Tumour Cells (CTCs) in NSCLC: From Prognosis to Therapy Design. Pharmaceutics.

[32] Quarta A., Gallo N., Vergara D., Salvatore L., Nobile C., Ragusa A., Gaballo A. (2021). Investigation on the Composition of Agarose–Collagen I Blended Hydrogels as Matrices for the Growth of Spheroids from Breast Cancer Cell Lines. Pharmaceutics.

